# “It is because we women do not have a voice to be heard” - perceptions of gender-based discrimination and its relevance to health: a qualitative study with women in Burkina Faso, Ghana and Tanzania

**DOI:** 10.1186/s12939-025-02719-5

**Published:** 2025-12-19

**Authors:** Verena Struckmann, Ruth Waitzberg, Clara Orduhan, Louise Charlotte Olimpia Junge, Sylvia Danso, Ali Sie, Peter Binyaruka, Daniel Opoku, Laurène Petifour, Swati Srivastava, Manuela De Allegri, Wilm Quentin

**Affiliations:** 1 Department of Health Care Management, Technical University Berlin, Straße des 17, Juni 135, 10623 Berlin, Germany; 2 Centre de Recherche en Santé de Nouna (CRSN), Nouna, Burkina Faso; 3 Ifakara Health Institute, Ifakara, Tanzania; 4 School of Public Health, Kwarme Nkuruhma University, Kumasi, Ghana; 5 Heidelberg Institute of Global Health, University Hospital and Medical Faculty, Heidelberg University, Heidelberg, Germany; 6 Chair of Planetary & Public Health, University of Bayreuth, Bayreuth, Germany

**Keywords:** Gender based discrimination, Maternal health, Gender inequality, Health equity, Sub-saharan Africa, Qualitative research

## Abstract

**Background:**

Gender-based discrimination (GBD) remains a pervasive determinant of health inequity for women globally, yet its systemic and culturally embedded forms in low- and middle-income countries are underexplored. This study explores women’s lived experiences of GBD in Burkina Faso, Ghana and Tanzania, highlighting how intersecting social and institutional norms influence access to health care, education, employment, financial resources and the resulting impacts on women’s health.

**Methods:**

Between February and May 2022, 17 focus group discussions and 32 in-depth interviews were conducted with 167 women across twelve regions in the three countries. Thematic analysis was employed to identify core patterns in how women perceive and navigate GBD in their daily lives.

**Results:**

Across all sites, participants conceptualized GBD as a normalized, systemic structure embedded in both public and private spheres. Women described GBD as omnipresent and internalized, upheld by cultural, religious, economic and educational norms that reinforced power imbalances, particularly in household decision-making. These structural constraints limited women’s access to education, employment, healthcare, and financial autonomy, and positioned them as both subjects of and gatekeepers to gendered hierarchies. GBD was identified as a key barrier to maternal care such as reproductive autonomy, with male dominance over contraceptive use, and pregnancy-related decisions. This lack of autonomy, compounded by institutional biases and sociocultural stigma, was perceived to contribute to delayed care, emotional distress, and adverse physical and mental health outcomes.

**Conclusion:**

The findings underscore the need for multisectoral strategies to address women’s health inequities. Efforts must focus on dismantling entrenched gender norms, enhancing women’s decision-making power, and ensuring institutional accountability for gender equity within health systems – not only in Burkina Faso, Ghana, and Tanzania.

**Supplementary Information:**

The online version contains supplementary material available at 10.1186/s12939-025-02719-5.

## Background

Gender-based discrimination (GBD) refers to any distinction, exclusion, restriction or unequal treatment due to gender that has the effect or purpose of impairing or nullifying the recognition, enjoyment or exercise of human rights and fundamental freedom [1]. It is a pervasive global issue with profound implications for women’s health and well-being, and it constitutes a major challenge for global health [2]. GBD arises from a complex interplay of structural, cultural, and institutional factors that shape power dynamics within societies, leading to unequal access to essential resources such as food, education, and healthcare—including maternal health services [3]. In low- and middle-income countries (LMICs), particularly in sub-Saharan Africa (SSA), GBD is reinforced by patriarchal norms, discriminatory laws, and socio-cultural practices that marginalize women and restrict their autonomy [2, 4–7]. These dynamics operate across multiple levels, household, community, and institutional, limiting women’s participation in decision-making and access to opportunities [8]. Discrimination and discriminatory practices in form of denying access to education, economic resources, and decision-making power are deeply rooted in both systemic barriers and internalized cultural and religious beliefs that reinforce women’s subordinate status [6].

The effects of GBD are particularly detrimental in the context of women’s health, including maternal health. GBD limits women’s access to quality healthcare, reduces their ability to make informed decisions about their care, and perpetuates to poor health outcomes [9–11]. Despite global improvements in maternal health, nearly 830 women still die daily due to pregnancy and childbirth-related complications, with SSA accounting for the majority of these deaths [12–14]. Women in SSA face multiple barriers that undermine their maternal health (in the perinatal period and beyond), including poor healthcare provider attitudes, economic inequities, low maternal education, poor access to health services and entrenched gender inequities [15]. Research indicates that GBD in maternal healthcare settings directly contributes to negative health outcomes, including delayed or inadequate care during labor, and influences future health-seeking behaviors [16].

While there is a growing body of research on GBD, much of it focuses on specific domains such as workplace discrimination [17, 18], gender-based violence [5, 19, 20], or health care-seeking behavior [21, 22]. However, there remains a notable gap in qualitative research that explores how women themselves define and experience GBD in the context of maternal health in SSA [16, 23]. Building on existing multi-country research on GBD, this study contributes by providing a qualitative exploration to understand women’s lived experiences and perceptions of GBD for designing interventions that address both structural discrimination and the everyday realities women face in accessing care.

### Conceptual approach for understanding GBD

We adopt the Johns Hopkins University Affiliate (JHPIEGO) Gender Analysis Framework (GAF) conceptual approach for understanding GBD across different domains of life [24]. The JHPIEGOs GAF is organized around four key domains that intersect with health outcomes:

1. ACCESS TO ASSETS: How gender relations affect access to resources necessary for a person to function in society. Includes tangible assets such as land, capital, and tools, and intangible assets such as knowledge and education.
2. BELIEFS AND PERCEPTIONS: How cultural belief systems or norms about what it means to be a man or woman in a specific society shape behavior, including participation, and decision-making capacity. They also facilitate or limit men’s and women’s access to healthcare, education, services, and economic opportunities.
3. PRACTICES AND PARTICIPATION: The norms that influence men’s and women’s behavior also structure the type of activities they engage in and their roles and responsibilities. For example, the capacity to participate in economic, political, and social activities, and their decision-making.
4. INSTITUTIONS, LAWS, AND POLICIES: How men and women differ in formal and informal rights, and how they are dissimilarly affected by policies and rules governing institutions, including the health system.

Power influences all domains, shaping who controls, accesses, and utilizes resources, as well as who makes decisions about their own bodies, household and family. It affects whether individuals can seize opportunities, exercise their rights, form associations, work, or participate in political life. Power also dictates how institutions, policies, and laws treat men and women differently [24].

This study aims to address the knowledge gap regarding women’s perceptions of GBD in SSA by exploring how they understand and perceive its manifestations and impact across various aspects of their lives in Burkina Faso, Ghana, and Tanzania. We focus specifically on how women perceive GBD as affecting access to healthcare, education, employment and financial resources, as well as how they navigate these challenges in their daily lives. The specific objectives are threefold:

1. To explore how women perceive, experience and conceptualize GBD.
2. To examine women’s perspectives on how GBD affects access to healthcare, education, employment, and financial resources; and how they navigate these challenges in their daily lives.
3. To analyze women’s perceptions of how GBD influences their health, with a particular focus on maternal health.

By providing insights into the lived experiences of women in SSA, this research seeks to contribute to the growing body of evidence on gender and health, and to inform more equitable policies and interventions that promote women’s autonomy and well-being.

## Methods

### Study design and population

This exploratory study used a qualitative approach and applied two data collection techniques: focus group discussions (FGD) and in-depth-interviews (IDI). The study was conducted with women in Burkina Faso, Ghana and Tanzania to develop an understanding of the individual perceptions of and experiences with GBD.

This study adopts a binary definition of gender, focusing on individuals categorized and socially perceived as women, reflecting the dominant socio-legal structures in SSA, which continue to frame gender within binary terms. While gender is a construct that links gender identity (a person’s individual identity) and gender expression (how individuals signal their gender to others), sex is a construct based on a cluster of anatomical and physiological traits. This distinction, emphasized in sociological and feminist theory and adopted in public-health guidance, highlights that while sex and gender interact, gender is produced and reproduced through social processes and everyday interaction [3, 25–27], and that scientific practices themselves can participate in constructing notions of biological sex [28]. For clarity, we use sex to report biological and medical measures and gender to refer to social roles, norms, and self-reported gender identity. Gender itself is a contested concept: its conceptualisation is political and may address or reinforce social inequalities [29]. In this paper we examine gender, as it is socially constructed and expressed [30]. This operational definition facilitates the analysis of discrimination as it is experienced and institutionalized within these contexts. Gender, as defined by [31], encompasses the socially constructed roles, expectations, responsibilities, and attributes assigned to individuals within specific cultural and social settings. These gender roles and expectations significantly shape how individuals perceive illness, seek medical care, and receive support from social care networks [32]. We focus on female gender-based discrimination because this is the most prevalent type of GBD in SSA [33]. The research, methods, and interview guides were approved by the Ethics Committee of Faculty VII Technical University Berlin (TUB) on 06^th^ December 2021 (project number: 20210917), the Ethics Committee of the Kwame Nkrumah University of Science and Technology (KNUST) in Kumasi on 16^th^ May 2022 (project number: CHRPE/AP/187/22) and the National Institute for Medical Research (NIMR) in Dar Es Salaam on 13th December 2021 (project number: NIMR/HQ/R0.8a./Vol.IX/3862) and the Nouna Health Research Center (CRSN) in Nouna on 10th December 2021 (project number: 2021-019-/MS/SG/INSP/CRSN/CIE).

### Research setting and sampling method

This study employed a three-pronged purposive sampling approach: First, the three countries—Burkina Faso, Ghana, and Tanzania—were selected based on two criteria: (1) the level of self-reported experiences of GBD, as measured by Afrobarometer data [34], and (2) representation of different SSA sub-regions. Afrobarometer conducts face-to-face interviews in the language of the respondent’s choice to measure self-reported experiences of GBD, their experiences with unfair treatment and perceptions of discrimination in various areas of life, including the workplace, public spaces, and within their communities [35]. Burkina Faso represented a high level of self-reported GBD, Ghana a medium level, and Tanzania a low level. Ghana, an Anglophone country in West Africa, has the lowest maternal mortality rate among the three countries, while Tanzania, an Anglophone country in East Africa, has the highest [35]. Despite this variation, all three countries continue to report maternal mortality rates exceeding the Sustainable Development Goal (SDG) 3 target of 70 maternal deaths per 100,000 live births.

Second, within each country, regions were selected to reflect variation in reported GBD levels. In Tanzania, Pwani (high incidence) and Morogoro (low incidence) were chosen based on regional data from [33]. In Burkina Faso, Nouna and Pâ were identified as high-GBD regions, while Dara and Babikolon were selected as rural areas with limited available data. In Ghana, a range of regions was included to ensure maximum variation: the Northern Region (Tamale, Savelugu, Kintampo), urban centers (Accra and Kumasi), and the Greater Accra Region (Ejisu), reflecting regional differences in GBD prevalence.

In Burkina Faso, gender inequality persists despite legal reforms. Customary practices continue to give men authority over land, income, and household decisions, leaving women with little control over resources [36]. Women’s economic contributions rarely translate into greater decision-making power; rather, they must negotiate their rights within patriarchal systems that expect obedience and submission, reinforcing dependence on men for access to healthcare and resources [37].

GBD in Ghana is deeply rooted in cultural and structural systems that perpetuate inequalities across social, educational, and health sectors. For example, cultural norms contribute to violence against women, lower female education, and limited political participation [38]. In schools, gendered classroom practices and stereotypical portrayals reinforce unequal power dynamics, while in healthcare, patriarchal attitudes portray women as incapable of making autonomous decisions [39]. Socio-cultural and economic barriers further restrict women’s ability to access high quality healthcare [40].

Similarly, in Tanzania, patriarchal structures uphold male dominance and normalize violence against women. Despite awareness of gender-based violence (GBV) among adolescent girls and young women, many accept abuse due to entrenched norms [41]. Men’s control over financial and reproductive decisions, combined with heavy domestic workloads and limited male involvement in maternal health, perpetuate gender inequities that compromise women’s well-being [42].

Third, participants were recruited using a combination of purposive and convenience sampling, facilitated by local co-authors [AS, SD, PB]. Eligibility criteria included women aged 19 to 54 years—encompassing the majority of the reproductive age span—who were able and willing to participate. Women were selected to ensure diversity in occupation (formal, informal, and domestic work), socio-economic status, and educational background. Recruitment took place in community settings such as women’s centers, local women’s groups, maternal health clinics, hospital maternity wards, and village meeting spaces. Participants were informed of their right to withdraw at any time or to decline to answer any question without consequence. The following information was provided to the participants as part of the informed consent process: If at any point during the interview there is even a slight indication that your safety or wellbeing may be at risk, the interview will be stopped immediately. You may experience some emotional discomfort, distress, or anxiety while discussing certain topics. Should this occur, the interviewer will pause or stop the interview and, if necessary, assist you in accessing appropriate professional support.

In each region we conducted one or two FGDs, and between one and five IDIs. Each FGD included 8 to 10 participants. To obtain deeper insights into individual experiences and to corroborate themes emerging from the FGDs, information-rich participants were selected for follow-up IDIs. This combination of FGDs and IDIs allowed for both breadth and depth in exploring the complex and context-specific manifestations of GBD in maternal healthcare [43]. Both the FGDs and the individual interviews were conducted by trained qualitative interviewers under the direct supervisions of those among our authors who are employed at institutions located in Burkina Faso, Ghana, and Tanzania. Local interviewers were all researchers with training in either sociology, anthropology, ethnography or qualitative health sciences and for this specific study, they were sensitized to issues pertaining to gender and GBD.

### Data collection tools

The JHPIEGO GAF clearly guided the development of the semi-structured interview guides for both the IDIs and FGDs by ensuring they explore the four domains as the guiding model for their design. Our entire team contributed to the development of the interview guide in a collaborative manner, so that perspective from multiple countries were all integrated in a single interview guide. While the logic of the interview guide remained consistent across countries, small adjustments were made to adjust specific country contexts. The questions address (1) access to assets and resources through inquiries about healthcare access, education and support systems; (2) beliefs and perceptions by asking about societal views on gender roles and discrimination; (3) practices and participation via questions on women’s roles and experiences in different contexts; and (4) institutions, laws, and policies by probing how health systems and social norms affect women differently. Overall, the framework shapes the guide to capture power dynamics influencing gender disparities in health and social participation.

It thereby offers a comprehensive lens for understanding individual and household, community, facility, district and program, national and system-level factors that shape GBD, and how GBD is expressed. It further provides a structure for organizing information about how gender inequalities affect health and access to care, making it a comprehensive and pragmatic guide supporting a systematic qualitative analysis of context sensitive factors for GBD. The focus group interview guide covered the following key topics (see Appendix 1 for the in-depth interview guide):

1. What does gender-based discrimination mean? Have you personally experienced or observed gender-based discrimination? In which context?
2. How do women’s positions differ from those of men in your society?
3. What does discrimination mean for women’s health? What factors affect your choice to access or seek health care, especially maternal health care?

In-depth narrative interviews were conducted with women who had participated in the FGDs in order to further explore their understanding of GBD and to elicit detailed accounts of their personal experiences. These interviews aimed to uncover how they perceive GBD, how and in which contexts it affects various aspects of their lives, with particular attention to its implications for women’s access to education, employment, financial prospects and health care, with a particular focus on its effects on women’s and maternal health consequences. A narrative interview approach was employed to gain deeper insights into the themes and experiences that had emerged during the FGDs, allowing participants to elaborate on their stories in a more personal and reflective manner [44].

The interview guide was translated to local languages in each country and then piloted before use (see Supplementary Material for the last version of the interview guide). Pilot interviews provided the interviewers an opportunity to contribute to shaping the interview guide actively, since small adjustments could be made to content and sequencing of questions, in light of their feedback on field experiences. The FDGs and IDIs were conducted in the respective local language (Twi, Kiswahili, French) audio-recorded, transcribed verbatim, and then translated into English. Participants were interviewed between February and May 2022. All participants received an information sheet explaining the study and data protection measures and signed an informed consent before the interviews started. The women received a small honorarium for their participation in the FDGs and IDIs.

### Data analysis

We conducted a thematic analysis to systematically examine the data [44], which involved familiarization with the transcripts, coding, and finally, building themes. The analysis combined deductive and inductive approaches. Deductive analysis followed the JHPIEGOs GAF framework, applying predefined codes to identify relevant patterns. Inductive analysis allowed for discovering emerging themes beyond the framework through open coding. The process involved familiarising ourselves with the data, generating and grouping codes, discussing and comparing them and refining themes to accurately reflect participants’ views.

We note here that although our sample was very heterogeneous, our analysis focused on extracting common patterns across the three countries. In line with the methodological postulations advanced by [45], we purposely engaged with an heterogenous sample, across countries and within countries, across women of different ages, ethnic backgrounds, educational levels, and socio-economic groups, as a means of identifying out of a diverse set of data a common core of set regularities and patterns [45]. Guided by our conceptual framework, our purpose was to understand how GBD plays out in everyday life across a series of settings, beyond the specificities that may affect these settings.

## Results

In total, 17 FGDs and 32 IDIs were conducted with 167 women across twelve regions in the three countries (see Table 1). Table 2 summarises the characteristics of the participants. Of the 167 participants, the majority was working in the informal sector (101), followed by the formal sector (39) and housewives (27). In terms of education, most had secondary (58) or college/higher education (42), while 25 had no education. The largest age group was 29–38 years (81 participants), followed by 19–28 years (48) and 39–54 years (38). Overall, the sample reflects diverse backgrounds in occupation, education, and age.

**Table 1:** Number of FGDs and IDIs per region within each country

Burkina Faso			Tanzania			Ghana		
(4 FDG/8 IDI)	FDG	IDI	(4 FGD/9 IDI)	FDG	IDI	(9 FGD/15 IDI)	FDG	IDI
Dara	1	2	Morogoro	2	4	Kumasi	2	3
Nouna	1	2	Pwani	2	5	Ejisu	1	2
Pâ	1	2				Kintampo	1	3
Babekolon	1	2				Accra	2	4
						Savelugu	1	1
						Tamale	2	2

**Table 2:** Demographic data of participating women

Characteristics	Number of Participants
**Occupation**	
Formal sector	39
Informal sector	101
Informal domestic worker	27
**Education**	
No education	25
Primary	42
Secondary	58
College or higher	42
**Age range [years]**	
19–28	48
29–38	81
39–54	38
**Total**	167

**Fig. 1:**
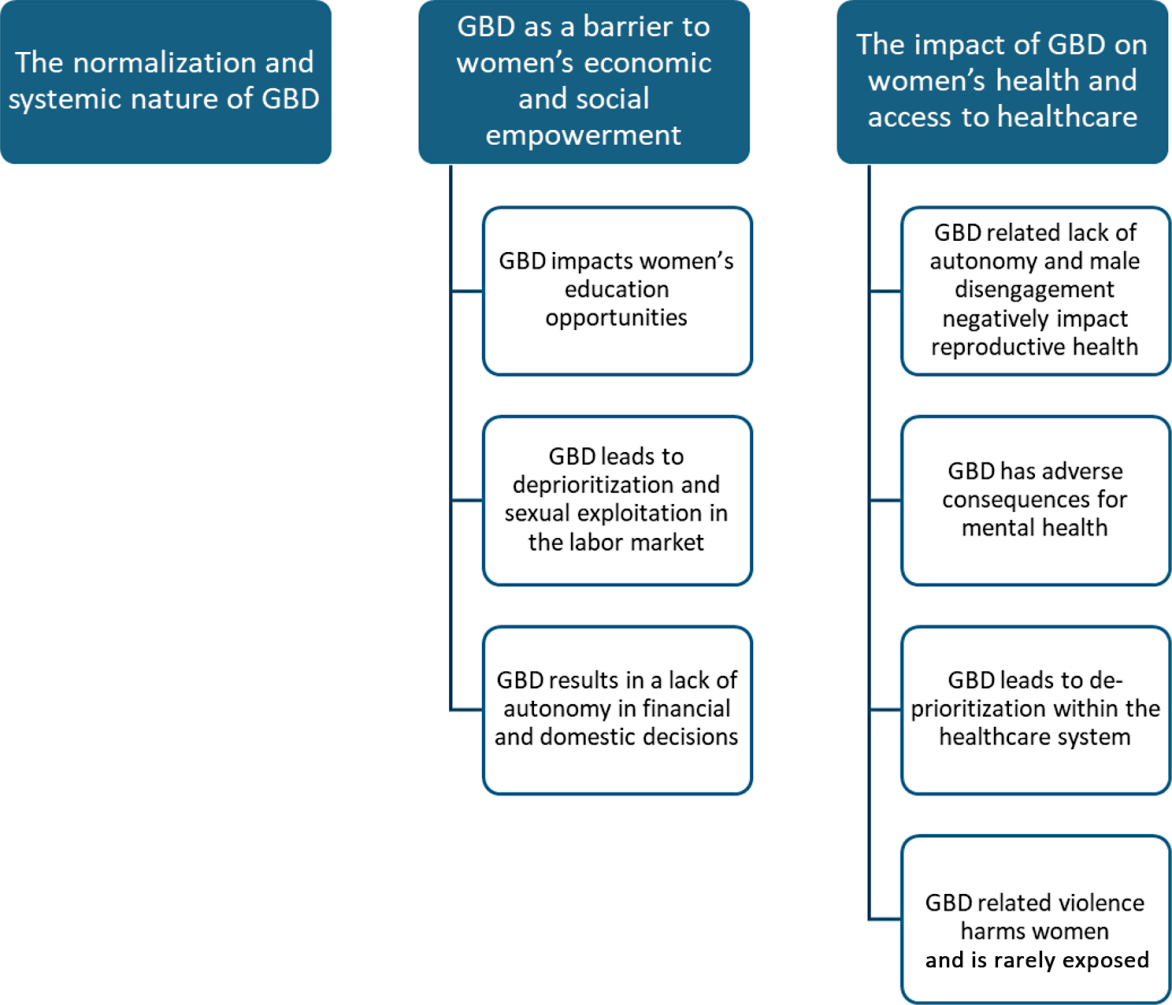
Themes and categories identified in the study

The results are presented thematically, drawing on participants’ narratives to illustrate the structural, social, and personal dimensions of GBD as experienced in Burkina Faso, Ghana and Tanzania (see Fig. 1 for an illustration of the findings). We identified three overarching themes based on and according to the predefined study objectives 1) The normalization and systemic nature of GBD; 2) GBD as a barrier to women’s economic and social empowerment and 3) The impact of GBD on women’s health and access to healthcare. Theme 2 and 3 are further clustered into categories, see figure below. We note here that the heterogeneity that went into our sampling strategy may not be apparent in our findings. This is, however, the result of a purposive decision, whereby we adopted maximum heterogeneity sampling to then identify a common thematic core across settings [45]. It was our explicit analytical intention to identify patterns that would be relevant at the continental level, beyond the country specificities. Moreover, our analysis did not capture any major differences across settings, confirming the validity of our initial approach to data. Intersectional analysis allows us to understand GBD not as a singular axis of inequality but as a phenomenon shaped through multiple, interlocking social positions, these include age, marital status, socioeconomic position, rural/urban location and educational status. Our cross-country sampling strategy, while oriented toward thematic convergence, nonetheless revealed that such structural intersections shape the salience, expression, and consequences of gender discrimination in lived experience.

### GBD is conceptualized as a systemic and normalized structure

Across all three countries, FDG and KII participants were very aware about the existence of GBD and perceived it as a negative and damaging phenomenon. Although the research question was framed broadly around health, the majority of FDG and KII participants predominantly focused on sexual and reproductive health as well as mental health, reflecting the domains in which GBD most directly affected their lives. They described GBD as a pervasive and deeply embedded feature of everyday life. Rather than viewing GBD as a series of isolated incidents, women conceptualized it as a systemic and normalized structure that shapes their roles, opportunities, and interactions across public and private domains. Their definitions were grounded in lived experiences and reflected the influence of cultural, religious, economic and educational norms existing. A central theme in women’s conceptualizations was the imbalance of power in decision-making, particularly within households.

*“It [GBD] affects us a lot because, men are the heads of the family their decisions are always seen superior to ours even when we are right. This goes a long way to affect our contribution to decision making” *[Woman from Tamale, Ghana].

This quote illustrates how GBD is understood as a denial of voice and authority, even in intimate and familiar settings and how gendered power imbalances intersect with marital status and household economic arrangements. Rather than being uniform, the meaning and consequences of men’s or husband‘s authority vary depending on women’s class position, caregiving burden, and access to education. All women also frequently described GBD as a negative behavior, omnipresent and inescapable, affecting them in all areas of life. Participants across all countries articulated how harassment and discrimination occur in virtually every sphere of life, emphasizing that women experience such treatment no matter where they go. This framing reflects a conceptualization of GBD as a constant and cumulative experience across multiple domains—societal, community, and personal.

Another prominent theme was the asymmetry in domestic roles and household authority. Participants across all three countries highlighted the entrenched power imbalances within the home, where male dominance is often unquestioned and that a husband has the authority to assign roles or spaces to his wife, while the wife lacks the reciprocal power to do the same. This underscores the structural and relational dimensions of GBD, where inequality is maintained through both cultural norms and gendered expectations within the family unit. Such accounts reveal how GBD is understood as a structural imbalance of rights and responsibilities, where women’s autonomy is constrained by social expectations. Importantly, many women articulated a sense of internalized devaluation, viewing their lack of power as naturally given, inevitable and unquestionable.

*“It is because we women are weak and do not have the means or the right. It is because we women do not have a voice to be heard”*. [Woman from Pâ, Burkina Faso]

This internalization of inequality shows that GBD is not only externally imposed but also psychologically embedded, shaping how women perceive their own worth and capabilities. A strong theme participants also emphasized across all countries was the social acceptance of gender hierarchies, highlighting the extent to which such structures are normalized within their communities. Several participants described how women’s subordinate status to men is widely accepted as a general truth across communities. This illustrates how GBD is conceptualized as a normative social order, reinforced by collective beliefs and practices. Intersectional theory helps explain this internalization: when women occupy positions simultaneously shaped by gender, limited education, rural residence, and economic precarity, the structural constraints become naturalized as personal deficiencies.

In some cases, GBD was also understood through the normalization of violence, particularly within marriage. This perception was especially evident in narratives from Tanzanian women, who described how physical abuse by husbands was often seen as a routine aspect of marital life. Such views reflected a broader community belief that violence in intimate relationships is an accepted and tolerable part of being married. This reflects a conceptualization of GBD as culturally sanctioned harm, where violence is not only tolerated but expected. Here, intersectionality draws attention to why violence is normalized more strongly in some contexts. Gender-based violence (GBV) intersects with socioeconomic dependence, legal precarity, and community norms that differentially position married, divorced, or economically insecure women. Our results showed that GBD is internalized by women and engrained in cultural belief systems. The majority of women adopt and justify discrimination, undervaluation, and mistrust towards themselves and other female peers. This internalized misogyny reinforces and legitimizes rigidly defined roles for women, fundamentally limiting their autonomy and societal status. A persistent manifestation of this inequity is the lack of decision-making power, particularly in domestic, financial, and bodily matters. Participants across all countries frequently described how cultural norms define women as caregivers and reproducers, often limiting their autonomy in health-related decision-making.

*„The doer of male order, she is supposed to obey everything she is told to do”*. [Woman from Morogoro, Tanzania]

Autonomy in one domain of women’s lives which is constrained by limitations in others, underscoring the intersecting nature of gendered constraints. Despite this subordination, women are paradoxically positioned as custodians of the household, expected to raise children while deferring to male decision-making. Social value is attributed through gendered contributions: men, as financial providers, fulfill the breadwinner archetype and are granted higher status; women, conversely, are defined by reproductive and caregiving roles, yet perceived as economic dependents.

Together, these narratives reveal that women conceptualize GBD as a multifaceted and deeply entrenched system—reinforced by social norms, internalized beliefs, and cultural practices.

### GBD reduces women’s access to resources and opportunities

The findings from objective 2 reveal how GBD systematically restricts women’s access to education, employment and financial autonomy, thereby limiting their agency throughout their lives. These structural inequalities not only constrain women’s economic and social opportunities but also shape their experiences within healthcare systems, particularly maternal health services (as described in section 3 of the Results).

#### GBD impacts women’s education opportunities

In all countries participants described a pervasive undervaluation of girls’ education. Early marriage, household responsibilities, and skepticism about investing in girls’ futures curtailed educational attainment and economic opportunities. Women who attempt to pursue education post-marriage may be perceived as challenging traditional gender roles, particularly described in polygynous households in Ghana.

*„Some of our parents are of the view that girl child education is not important. Even if they will cater for your education, at a point you will be withdrawn for marriage. Some men will promise you school after marriage but after marriage, it ends there and you are left with nothing to do. If the girl continues to remind the man about his promise to get her back into school, the man gets angry and sees it as she rivaling other wives. As a result of this if there is a vacant position, women cannot compete with their male counterparts due to their low educational level“*. [Woman from Savelugu, Ghana]

The majority of women are often undervalued even within their own families, where their roles are predominantly perceived through the lens of domestic responsibilities and childbearing. This perception significantly contributes to a lack of investment in their health, education, and long-term development. In some contexts, families facing financial constraints prioritize the education of boys over girls, viewing the latter as temporary members of the household who will eventually be married off. As a result, daughters are frequently denied educational opportunities, as their future contributions are not seen as yielding sufficient returns to justify the cost. This disparity contributes to the systemic prioritization of men and boys, particularly in access to education and health services.

*“A man will be given everything that both of you deserve, for example in the education sector, a man will be given first priority than a woman. In health sector too, a man will be given first priority for the welfare of the family so he has to be healthy and fit for the provision of the family”*. [Woman from Pwani, Tanzania]

This statement shows how rooted gender norms and the undervaluation of girls’ education limit women’s access to resources and opportunities are. The resulting lack of resources compounds the undervaluation of women with regard to health care and limits their ability to advocate for their own well-being. This also illustrates how gender norms intersect with the social expectation that men act as economic providers, a role tied to social status and familial legitimacy. Intersectional analysis therefore clarifies why women’s access to education and health is shaped not only by gender identity but also by socially constructed household roles and economic responsibilities.

#### GBD leads to deprioritization and sexual exploitation in the labor market

Across multiple study sites in Burkina Faso, Ghana and Tanzania, women consistently reported being deprioritized in the labor market, often as a direct consequence of GBD. Pregnancy, in particular, was perceived not only as a biological condition but as a professional liability, resulting in discrimination and job insecurity. The majority of women described being overlooked for opportunities or dismissed outright due to assumptions about their capabilities during pregnancy. In some cases, fear of job loss was so pronounced that women deliberately avoided pregnancy or even considered terminating pregnancies to retain employment, highlighting the extreme pressures faced in reconciling reproductive choices with economic survival. Institutionalized gender norms shaped hiring and promotion practices, frequently relegating women to domestic roles or lower-status jobs regardless of their qualifications. Even when equally or better qualified, women were passed over in favor of male candidates.

*„I just remembered something that happened to me about 3 months ago at a top university in Kumasi. I had submitted an application there about a year ago but they hadn’t gotten back to me. When they called me, I was already pregnant. I knew I had the strength to do the job but in the midst of everybody, they told me, but you are pregnant and I told them, yes but I can do the job that is why I came when you called me. I just need the opportunity to show that I can do it. I thought they were going to give me the position but they haven’t called me since then. It got me thinking whether it meant that then as women when we get pregnant, we can’t do anything but rely on our husbands or friends or beg people to survive. It means that the society does not give pregnant women the chance to work in order to make ends meet”*. [Woman from Suntreso, Ghana]

Intersectionality illuminates how pregnancy stigma operates at the intersection of gender, reproductive status, and employment precarity. Women in informal or low-wage sectors face heightened risks because their class position and limited labor protections amplify gendered discrimination. This sense of exclusion extended beyond pregnancy to broader GBD, often manifested through occupational segregation and limited access to traditionally male-dominated sectors. Many women described being fully capable of performing tasks assigned exclusively to men but were nonetheless forced to abandon such opportunities due to prevailing discriminatory norms. Stereotypes about leadership capabilities further constrained women’s advancement in the formal sector. Gendered assumptions about competence and authority contributed to the systematic undervaluation of women’s leadership potential, as decision-makers often preferred men for leadership roles based on the erroneous belief that women were inherently less suited to lead. These structural barriers intersected with more coercive and exploitative dynamics, particularly in the form of sexual exploitation within both formal and informal employment contexts. Several participants recounted experiences where access to employment was made conditional upon acquiescing to sexual demands from male employers or intermediaries. In such cases, women’s bodily autonomy was directly leveraged as a condition for economic survival, with refusal often resulting in job loss or exclusion from employment altogether. The normalization of what some participants called “sexual corruption” underscores how systemic coercion operates through deeply entrenched gender hierarchies.

*„You give sexual corruption if you need any kind of a job even if it’s a job at the bar. If you go and ask for a job in the office the boss will ask you for sex, and others will ask you for it and thereafter you might not even get the job“* [Woman from Pwani, Tanzania].

Such practices are particularly harmful in resource-constrained settings where women lack financial autonomy and are disproportionately vulnerable to poverty. Economic dependence on male partners or employers, coupled with social stigmas around female sexuality and labor, further entrenches exploitative dynamics. In some cases, the denial of economic support by male partners was cited as a trigger for survival strategies that involved transactional or exploitative sexual relationships.

*“There are some women who will go to men, do sexual vagrancy because her husband refuses to give her money to take care of her child […]”*. [Woman from Mossi, Burkina Faso]

#### GBD results in a lack of autonomy in financial and domestic decisions

As a result of GBD limiting women’s access to education and opportunities in the labor market, women across all countries reported substantial financial dependence on husbands or parents. In addition, they often lack control over their own earnings. Even when engaged in paid work, cultural norms deny them economic autonomy. This dependency frequently leaves women with little control over their own lives. In some cases, women rely entirely on their parental families for financial support, as their spouses provide little or no assistance, highlighting the severity of their economic vulnerability and lack of agency.

*“Once you are married to a man, everything you earn belongs to the man”*. [Woman from Nouna, Burkina Faso]

This lack of financial autonomy was closely linked to limited decision-making power in the domestic sphere. Men determine how the tasks of housekeeping and domestic life should be conducted, even though these tasks are considered to be the responsibility of women.

*“The work of woman is to stay at home to take care of family; the husband looks for daily bread, which means will torture her knowingly that [he] is the bread earner and ‘everything here at home, and also family leader; so, you will live the way I want”*. [Woman from Morogoro, Tanzania]

### GBD influences access to health care and negatively impacts women’s health

Across all countries the majority of participants explained a complex interplay of structural and interpersonal dynamics that reinforce gendered power imbalances, particularly in the domains of fertility and reproductive rights. They described how systemic deprioritization of women’s health within healthcare institutions, coupled with male disengagement in reproductive and maternal health, undermines access to care. Additionally, barriers to disclosing experiences of GBD and GBV further exacerbate health vulnerabilities. These themes underscore the pervasive influence of gendered norms and institutional neglect in shaping women’s health trajectories. Furthermore, lack of autonomy over reproductive decisions, persistent emotional distress, and delayed or substandard care are deeply rooted in the power imbalances and discriminatory practices outlined in earlier sections. Applying intersectionality to women’s access options to health care highlights how gender is compounded by marital status, economic insecurity, cultural norms, and provider-level discrimination.

#### GBD related lack of autonomy and male disengagement negatively impact reproductive health

Across study sites, GBD was consistently described as a lack of autonomy and control over decisions related to women’s bodies, health, and reproductive lives. In general participants highlighted how entrenched gendered power imbalances, particularly within intimate relationships, limited their ability to make independent choices about sexual activity, contraceptive use, pregnancy, and access to health services. Male partners were frequently identified as the primary decision-makers, with women often needing permission or financial support to seek care. These dynamics not only undermined women’s reproductive agency but also heightened their vulnerability to coercion and violence. Women across all countries frequently described how societal norms reinforce male authority over reproductive decisions, leaving them with little room to negotiate or assert their preferences.

„*It [my health] worries me but what can I do about it? I feel that if I were a man, we will have a book with responsibilities stated for everybody. But since I am a woman, I can’t say anything, even with respect to getting pregnant or not”*. [Woman from Ridge, Ghana]

Male disengagement in reproductive and maternal health further compounded these challenges. Many women reported that their partners lacked awareness of, or interest in, maternal health needs and were often unwilling to provide financial or emotional support. This disengagement created significant barriers to accessing care, particularly in contexts where women were financially dependent on their husbands.

*“There are even men who tell you that instead of spending a lot of money on their wife’s health, they would rather marry a new wife”.* [Woman from Nouna, Burkina Faso]

Others described the burden of having to convince their partners to support clinic visits or pay for essential services.

*“There, a lot of things that they don’t know, for example clinical costs, when you’re going for clinic, there are lab test that have to be carried out like ultra-sound in order to see position of the baby. Now most men wouldn’t understand this because they hardly attend clinic sessions”*. [Woman from Pwani, Tanzania]

A recurring theme in the data across the countries is the perception that men tend to emotionally and practically distance themselves from pregnancy-related responsibilities. This disengagement manifests as men adopting a detached or indifferent attitude during their partner’s pregnancy, as if the pregnancy is not their concern or burden to share. Such behavior contributes to a broader pattern in which GBD, compounded by men’s lack of involvement and women’s financial dependence, systematically erode women’s reproductive autonomy. In Burkina Faso and Ghana men especially refused financial responsibility during pregnancy. This dynamic ultimately restricts women’s access to critical health services, reinforcing inequities in reproductive health outcomes.

#### GBD has adverse consequences for mental health

There are multiple pathways through which GBD exerts adverse mental health outcomes, notably emotional dysregulation, distress, psychosocial withdrawal, and somatic symptoms. In particular participants from Ghana and Tanzania described behavioral changes after experiencing GBD.

*“I told you earlier that psychologically I was going off at a point and how it [GBD experience] affected my work and how I became abusive and defensive at a point. […] I also stay with my mum so I decided to turn a blind eye to some things, concentrate on my side business which is catering and I also sing in the church so I decided to get more involved at church”*. [Women from Ejisu, Ghana]

Intersectionality highlights that emotional and psychological impacts accumulate differently depending on women’s social location. For younger and unmarried women, or those with limited economic options, the mental health consequences of GBD are intensified by overlapping forms of marginalization. Women’s own acceptance and internalization of GBD influences their role in society and their own self-perception, often leading to them undervaluing their own lives. Internalized GBD and the lack of emotional and institutional support had psychosocial impacts at multiple levels and potentially results in mental health difficulties.

*“You should never argue with your husband and whatever happens just be patient, you have no option other than remaining silent and you can’t do anything, so this happened to me too and I wished like committing suicide but I didn’t know how”*. [Woman from Morogoro, Tanzania]

Moreover, chronic stress and somatization were frequently reported. Some participants described symptoms such as persistent fatigue, unexplained weight loss, and cardiovascular complaints attributing these to prolonged emotional strain and the social stigma surrounding public disclosure of GBD.

*„If you are depressed because of discrimination by the spouse, you can lose weight and when they see you they think you are sick when you are fine. They do tests with no results, you always have heartaches”*. [Woman from Pa, Burkina Faso]

#### GBD leads to deprioritization within the healthcare system

The majority of participants consistently reported that gender significantly influenced timely access to healthcare services and increased barriers in accessing maternal healthcare. Women across all three countries, particularly those unaccompanied by male partners, experienced delays or received lower-quality care. They were often deprioritized in clinical settings, subjected to verbal mistreatment, or denied prompt attention. Healthcare providers, especially nurses, tended to respond more rapidly to male patients than to female patients, reflecting a gender bias in treatment prioritization. Additionally, men and married women with children were given preferential treatment over unmarried women or those without children, indicating that marital status and motherhood further influenced the level of discrimination encountered by women.

*“Different types of treatment among women for those who attend clinic with their partners and those that attend clinic alone. You find a woman has gone to the hospital early in the morning and she is in a queue ready to be attended but if another woman with her partner comes the other one who came earlier she will be left unattended”*. [Woman from Pwani, Tanzania]

These accounts highlight how entrenched gender norms and structural discrimination within healthcare systems across all three countries delay access for women, reinforcing unequal health outcomes. Intersectionality helps explain why discrimination is patterned by marital status, partner presence, perceived respectability, and motherhood. These interacting social markers determine which women are deemed deserving of care. Some women mentioned that these problems disappear when they sought private instead of public hospitals, provided that they can afford it. There, women pay for the treatment out-of-pocket (OOP) or through a voluntary health insurance (VHI) and resources are not as limited as in public hospitals. The need of prioritizing resources is reduced and women perceived less discrimination in these settings.

*“If I see I have enough money to go private then I will go there private I know when they receive the money, […] I receive good caring”*. [Woman from Morogoro, Tanzania]

#### GBD related violence harms women and is rarely exposed

The narratives shared by women in Burkina Faso and Tanzania underscore the pervasive exposure to violence that women endure, particularly within intimate partner relationships. Participants from both countries and in particular from Tanzania described how entrenched gender norms and power imbalances legitimize male dominance and normalize even the most extreme forms of violence. In one account, a woman described witnessing severe violence inflicted by a husband on his wife, followed by a community-driven cover-up and bribe.

*“[…] I witnessed, when I was living in a renting house; my landlord had been in conflict with his wife for a long time and ever done her bad thing; he cut ears, private part which demarcate vagina and anus (in front and back), mother [wife] was badly hurt. But she was told to say that was invaded by bandits when attend to hospital for treatment, and they will buy her ten pairs of Kenyan Khanga [traditional cloth]. She is still alive to date”.* [Woman from Morogoro, Tanzania].

Women from Tanzania who attempt to formally report their partner’s violent behavior often face increased risks rather than protection. Study participants revealed that reporting abuse can provoke retaliation from the perpetrator, frequently escalating the severity of violence as men respond with heightened anger to the perceived loss of control or public embarrassment. Many women expressed fear of social exposure and public shame as significant barriers to seeking help or medical care. This fear is deeply intertwined with cultural norms that emphasize women’s roles as submissive, discreet, and silent, discouraging them from disclosing abuse. Consequently, many women hesitate to access nearby healthcare services or support systems, concerned that revealing their experiences would not only bring personal disgrace but also dishonor their partners.

*“From society perspective a woman is also supposed to bear and keep silent on her marital affairs for the sake of her family and her children”*. [Woman from Morogoro, Tanzania]

These findings illustrate how sociocultural norms, institutional stigma, and internalized beliefs surrounding GBD create significant barriers to disclosure, ultimately limiting women’s access to healthcare and negatively impacting maternal health outcomes. The pervasive fear of judgment or dismissal by healthcare providers and institutions discourages women from seeking care or reporting abuse, reinforcing cycles of silence and inadequate maternal support.

## Discussion

To our knowledge, this is the first multi-country qualitative study that analyses women’s own definitions and lived experiences of GBD in the context of maternal health in SSA. It offers a nuanced and multi-layered exploration of how women in Burkina Faso, Ghana and Tanzania conceptualize and experience GBD, and how these experiences shape their access to education, employment, financial prospects and health care, as well as their maternal and mental health consequences.

Although the sampling strategy intentionally captured countries with differing self-reported GBD prevalence (high in Burkina Faso, medium in Ghana, low in Tanzania), women’s narratives across all sites revealed strikingly similar conceptualizations of GBD as systemic, normalized, and deeply embedded in everyday life. Women’s narratives underscore a developed consciousness of how patriarchal norms and institutionalized gender hierarchies that operate at societal, community, and interpersonal levels, constrain their autonomy, limit their access to education, employment, and health services, and expose them to systemic violence and marginalization. These structures are not only externally enforced but also internalized, contributing to the normalization and acceptance of discriminatory practices. The consequences of GBD are far-reaching, affecting women’s social roles, economic participation, and particularly their maternal and mental health. These findings correspond closely with the JHPIEGO GAF across its four domains (1. access to assets, 2. beliefs and perceptions, 3. practices and participation, and 4. institutions, laws, and policies), illustrating how power dynamics systematically restrict women’s resources, roles, and access. Beyond the JHPIEGO GAF, the results highlight profound internalized psychological impacts of GBD, such as the acceptance of devaluation, normalization of violence, and fear and stigma that hinder disclosure and access to care. These aspects underscore critical dimensions of gender inequity that extend beyond those explicitly addressed by existing frameworks.

Albeit the deep rooted GBD, women perceive it as harmful and negative, and imply that they would rather revert this phenomenon. These insights point to the urgent need for structural and systemic interventions that address the root causes of GBD and promote gender equity across all domains of life.

Intersectionality, as articulated by [46] positions gender not as a single axis of disadvantage but as a relational system that interacts with other structures of power such as age, marital status, class position, rurality, and access to education. Applying an intersectional lens to our study, it underscores that women’s experiences of GBD are not uniform and that there is no single axis of disadvantage. For example, younger, unmarried, less-educated, or economically insecure women frequently described more intense constraints on autonomy and more severe barriers to accessing health care. This supports [47] argument that intersectionality is essential for explaining within-group heterogeneity and for understanding why ostensibly similar gendered norms produce differentiated outcomes across women’s lives.

### Conceptualizations of GBD

This study aligns with and expands on a growing body of literature [48–50] that conceptualizes GBD not merely as a set of isolated incidents, but as a structural and systemic phenomenon embedded in everyday life. Across all three countries, women described GBD as an omnipresent force shaped by patriarchal values, cultural expectations, and institutionalized norms. Their accounts highlight the need to understand GBD not only as a legal or policy issue but as a lived reality shaped by intersecting layers of power and inequality. This aligns with existing literature that identifies patriarchy as a system that institutionalizes female subordination through restricted access to education, employment, and healthcare, while fostering internalized misogyny [51, 52].

Discrimination in the labor market further entrenches inequality, as women reported facing unequal pay, limited opportunities for advancement, and punitive maternity policies [52, 53]. These narratives illustrate how GBD in the labor market is not limited to hiring and promotion biases but is embedded within broader socio-economic structures that reinforce exploitation and perpetuate cycles of inequality.

Internalized oppression emerged as a powerful mechanism of GBD. Many women viewed their subordinate roles as natural or inevitable—an outcome consistent with [19], who argue that gendered socialization sustains unequal power dynamics. In Ghana, for example, women are expected to be respectful, dutiful, and serviceable to their husbands. Challenging abuse is often interpreted as a disruption of male authority [54, 55].

This internalization intersects with class, marital status, and age, as younger, unmarried, or less-educated women often face more intense scrutiny and restriction, reinforcing gender hierarchies not just across but within groups of women.

### Barriers to access and utilization of resources and opportunities

GBD acts as a foundational barrier to women’s access to education and employment. Participants across all three countries emphasized that discrimination begins early, with families and communities devaluing girls’ education in favor of early marriage and domestic responsibilities. These early disadvantages reduce educational attainment, inhibit labor market participation, and perpetuate intergenerational cycles of inequality. This aligns with prior studies showing how unequal access to education limits women’s economic independence and undermines long-term health and socioeconomic outcomes [19, 56].

Restricted education and employment opportunities often result in financial dependency, which emerged as a central theme. Many women in all three countries reported lacking control over their own earnings, even when employed. This dynamic facilitates male dominance in household decision-making, particularly in relation to reproductive and health-related choices. Women’s autonomy over contraception, pregnancy, and access to care was frequently curtailed, with some experiencing coercion, including forced prostitution or transactional sex.

Discrimination within the labor market operates on multiple levels. Pregnancy and motherhood were often treated as liabilities, exposing women across the countries to workplace insecurity, harassment, and coercive sexual demands. These practices reflect how structural violence operates at the intersection of gender, poverty, and economic precarity.

Intersectionality was evident in how these challenges disproportionately impacted unmarried, younger, or less-educated women. Structural barriers in education, employment, and healthcare systems are mutually reinforcing, limiting agency while increasing vulnerability to exploitation and abuse. These findings are consistent with broader regional evidence [57, 58], underscoring the need for interventions that address systemic and intersecting forms of marginalization.

### Health implications of GBD: maternal and mental health outcomes

GBD is reflected in everyday behaviors and attitudes regarding health. For instance, men frequently detach from women’s health or maternal care, reinforcing a broader societal perception that women’s health is of lesser value. The health consequences of GBD are far-reaching, particularly in the domains of maternal and mental health [4, 8]. While prior research has highlighted the role of internalized oppression, our findings underscore how systemic discrimination and economic dependency further constrain women’s ability to access essential health care services.

Across all three countries, women reported that male partners avoid their responsibilities during pregnancy, take little interest in women’s health and control household finances, regardless of whether women had independent earnings. Financial barriers, such as the inability to afford transport or pay for health care services, are compounded by male control over household resources, consistent with earlier studies [59–61]. Women often depend on male partners for permission, accompaniment, or financial support to seek care [41, 62–64].

Crucially, GBD is not only a social issue, but it is also deeply embedded in healthcare systems, where it is often perpetuated by caregivers and results in discriminatory treatment of women. As a result, women received differing levels of healthcare depending on their accompaniment (i.e., whether they have a partner or not), their marital and reproductive status and their economic situation, which was often highlighted by Tanzanian participants in particular. When men refuse support (financially or by accompanying their partners to appointments), women face additional obstacles or outright neglect. In the context of maternal health, which is exclusively aimed at women, this is particularly troubling, as it can lead to serious consequences for both mother and child.

Our findings show that gendered discrimination within healthcare settings, including disrespect, denial of services, and longer waiting times, directly limits women’s access to essential services. These discriminatory encounters are particularly pronounced in maternal and antenatal care [62]. Moreover, our findings draw attention to intra-gender hierarchies that shape access to care. Married women and mothers were often treated more favorably than unmarried or childless women, revealing how marital and reproductive status intersect with gender to stratify healthcare experiences [65]. These inequities reflect that even within the same gender, there are hierarchies that intersect with social class, age, and marital status.

Financial dependence and cultural norms that value male dominance create conditions for exploitation and violence, while these intersectional disadvantages discourage women from reporting abuse and from seeking family planning services or health, in particular antenatal care [4, 19, 56, 66–70].

Our findings further support existing literature indicating that GBD contributes directly to GBV, including physical, sexual, and emotional abuse [66–68]. Moreover, community responses that frame violence as private marital matters reflect broader societal complicity in silencing victims and obstructing timely and appropriate interventions. According to the experiences shared by the women in all three countries there are multifaced barriers to disclosing GBD and GBV, highlighting how sociocultural norms, institutional responses, and internalized stigma converge to silence women’s experiences. The normalization of gendered violence, particularly intimate partner violence, was described as a culturally sanctioned expression of male authority. This aligns with [27] concept of “normative violence”, and supports findings from [55] and [68], who note that social tolerance of violence against women is a key mechanism through which GBD is perpetuated. These dynamics discourage women from reporting violence or seeking family planning and maternal health services, reinforcing cycles of harm [4, 19, 56, 66–70].

A central theme emerging from our study is the lack of autonomy over their own bodies, and over reproductive decisions. Women across all countries reported being denied the right to make choices about sexual encounters, contraception, pregnancy, and antenatal care, experiences that directly undermine reproductive rights and increase the risk of adverse health outcomes. These findings are consistent with previous research showing that male-dominated decision-making in reproductive health contributes to delayed care, unintended pregnancies, and complications during childbirth [71].

Closely intertwined with these structural barriers is the reinforcement of rigid gender roles through GBD, which assigns women primary responsibility for caregiving and reproduction while simultaneously limiting their autonomy in decision-making. These roles, as articulated by women in Tanzania and Burkina Faso, are not only externally imposed but also socially sanctioned and often internalized by women themselves. The normalization of these roles further entrenches disparities in access to care and exacerbates health vulnerabilities.

In addition to undermining maternal health, mental health has emerged as a critical, yet often overlooked dimension of GBD. In many communities, mental health issues are misunderstood or dismissed, compounded by a general lack of awareness and education about mental health, leading to social exclusion and discourages individuals from seeking support [72, 73]. Many women described experiencing chronic stress, emotional exhaustion, psychosomatic symptoms, and in some cases, suicidal ideation. These mental health impacts were shaped not only by external violence but also by internalized discrimination, with women frequently blaming themselves for their suffering or accepting mistreatment as normal. This aligns with findings from [31], who emphasize that the psychological impacts of GBD serve both as a consequence of patriarchal structures and a mechanism for their perpetuation. Despite the severity of these outcomes, mental health remains under-addressed in both policy and services, underscoring a critical gap in the implementation of gender-sensitive health interventions and equitable resource distribution.

## Policy implications

Improving maternal and mental health outcomes for women in Burkina Faso, Ghana and Tanzania requires much more than targeted health interventions, it demands a full-scale transformation of the health system and broader structural and societal reforms addressing the root causes of GBD.

Concerning the health system, this study reinforces the need for expanding Universal Health Coverage (UHC), including the provision of free reproductive, maternal, and child healthcare, to reduce financial dependency and improve access to essential services [74]. However, improved access must be accompanied by system-wide long-term reforms to become more inclusive and equity-oriented, addressing the layered and intersectional nature of discrimination that women face [75]. These include combatting mistreatment in healthcare settings, integrating mental health services into maternal care, and ensuring dignified care regardless of marital or reproductive status.

Beyond the health sector, the findings point to the urgent need for structural reforms that address the root causes of GBD across all three countries. Patriarchal norms, economic dependency, educational disparities, and sexual abuse continue to shape women’s access to resources and opportunities.

Legal protections against GBV must be strengthened and enforced, while labor market policies should promote gender equity and protect women from discrimination, particularly during pregnancy and motherhood [76]. Additionally across the three countries, sexual harassment and abuse must be addressed through stronger reporting mechanisms and stricter enforcement of the law (e.g. punishment) in all institutions, including public hospitals. Education systems must also play a transformative role by challenging gender stereotypes and supporting girls to remain in school. Gender sensitive cross-regional advocacy and awareness campaigns that address social injustices, explain GBD, and involve the broader community, including men, can be effective. Finally, this study highlights the importance of targeted interventions addressing GBD at early stages to disrupt intergenerational cycles of inequality. Policies that promote girls’ education, reproductive autonomy, and economic empowerment can play a pivotal role in reducing violence and improving long-term health outcomes [77]. By centering women’s voices and lived experiences, this research underscores the need for comprehensive, cross-sectoral strategies to dismantle the structural foundations of GBD.

Embedding intersectionality into health system strengthening aligns with the WHOs recent emphasis on sex- and gender-responsive standards such as SAGER and GATHER [78]. Intersectional analysis can guide the design of equity-oriented indicators that move beyond binary sex disaggregation to capture the multiple and overlapping structures of power that shape women’s access to respectful, timely, and high-quality care. Integrating such frameworks into monitoring and evaluation would allow health systems to better identify which groups of women are most disadvantaged and why, thereby supporting more targeted, just, and context-sensitive interventions.

## Limitations

Our study has several limitations. First, the results are based on experiences with three different cultures, norms, societies and health care systems. Specifics of these norms, societies or health care systems are reflected in the experiences of the participants in the results of this analysis. This has to be considered carefully when applying the results to other countries and health care systems in SSA and beyond.

Second, the interviews were conducted by different individuals in each country and interviewers might be biased due to their personal experiences. This may result in different reactions and interactions with participants during the interviews [79]. However, at the time of the study COVID-19-related travel restrictions made it impossible to have one principal interviewer for all fieldwork. Yet, there are several advantages in working with multiple local researchers, as their deep understanding of the cultural and societal background of the women and the ability to speak the local language facilitates a closer connection with the interviewed participants. Furthermore, we held detailed online briefings and training sessions with the interviewers before the actual interviews took place.

Third, in the context of this paper gender is solely considered and assessed in its binary sense. To allow for insights into additional non-binary identities additional research is needed.

Finally, the FGDs included different groups of participants with a wide range of ages per group, which could potentially result in an unbalanced distribution of power, respect, and societal status resulting in response bias. For instance, younger women might feel intimidated and hesitant to speak freely in the presence of elderly women [80, 81]. Future studies, including different age groups, different levels of education, employment or occupation, and religion, may add a wider range of perspectives and depth of experiences, enriching the discussion and data.

## Conclusion

The findings of this study highlight the profound impact of GBD on women’s health and safety, in particular maternal healthcare, emphasizing the interconnections between social inequality, reproductive rights, healthcare access, and mental health. The evidence presented underscores the urgent need for interventions aimed at empowering women, dismantling patriarchal structures, and addressing the systemic biases within healthcare systems. Furthermore, fostering male involvement in reproductive health, raising awareness about GBD, and promoting economic independence for women are essential steps toward mitigating the harmful effects of gender inequality. To improve women’s health and well-being, particularly maternal and mental health, in contexts where GBD persists, a comprehensive approach is needed that includes both individual empowerment and structural reforms.

*“An end must be put to gender-based discrimination in the family, at work. It is everywhere even in hospitals. Someone can just see you and by your appearance will discriminate against you”. [Women from Suntreso, Ghana]*

## Electronic supplementary material

Below is the link to the electronic supplementary material.

Supplementary Material 1

## Data Availability

Anonymised data will be made available by the corresponding author upon a reasonable request.

## In-Depth Narrative Interviews – Interview Guideline:

Thank you very much for being part of our project MeasureGender. You already took part in our Focus Group Discussion. So, you know that MeasureGender aims to understand individual perceptions and views of women in Sub-Saharan Africa about gender-based discrimination. Even if you already told something in the FGD please do not hesitate to tell it again. There are no wrong answers. Everything you say will be kept confidential.

You can find all the general information in relation to the study design, data collection, and data storage on the information sheet.

Is there any question for now? If there are no more questions, I would like to start right away. But please feel free to ask any upcoming question at any point of the interview. I will now start the audio-record to begin with the first question of the interview.

*[GBD = gender-based discrimination]*

Interview Questions

In the FGD you told us about your personally experienced and/or observed situations of GBD *[refer to the experiences that were mentioned by the women].*

1. Could you go more into detail and tell me about these experiences? *[If there are different experiences, mention all of them, one after the other.]*
  - How do these experiences affect your health and your health behaviors?
  - What expectations do you have when seeking maternal health care services?
  - Besides pregnancy-related health issues, can you tell me about health issues women are affected by more than men?
  - What are potential reasons for these health issues?
  *Objective: To understand experiences with GBD in detail, and to uncover links to health. Women are encouraged to tell their stories in detail, we just want to guide them.*
2. Is there anything else you want to add or something that we have not discussed but seems important to you?
  For any question section there can be used the following questions in order to receive additional clarification in the end:
  - Can you expand a little on this?
  - Can you tell me anything else?
  - Can you give me some more examples?

## Abbreviations

CRSN: Nouna Health Research Center
FGD: Focus group discussions
GAF: Gender Analysis Framework
GBD: Gender-based discrimination
GBV: Gender-based violence
IDI: In-depth-interviews
JHPIEGO: Johns Hopkins University Affiliate
KNUST: Kwame Nkrumah University of Science and Technology
LMIC: Low and middle-income countries
NIMR: National Institute for Medical Research
OOP: Out-of-pocket
SDG: Sustainable Development Goal
SSA: Sub-Saharan Africa
TUB: Technical University Berlin
UHC: Universal Health Coverage
VHS: Voluntary health insurance
