# Supplementary material for: “It is because we women do not have a voice to be heard” - perceptions of gender-based discrimination and its relevance to health: a qualitative study with women in Burkina Faso, Ghana and Tanzania
Source: Int J Equity Health. 2025 Dec 19;25:30. doi: 10.1186/s12939-025-02719-5 (PMC12853705; doi:10.1186/s12939-025-02719-5)
Supplement: Supplementary file 1 — Supplementary Material 1 [file 12939_2025_2719_MOESM1_ESM.docx]

Supplementary materials

Appendix 1

**1 - Focus Group Discussions – Interview Guideline:**

Thank you very much for being part of our project MeasureGender. MeasureGender aims to understand individual perceptions and views of women in Sub-Saharan Africa about gender-based discrimination. Today we are going to conduct a Focus Group Discussion which means that I will ask some questions and everyone, who feels like having to give some input, can answer them. A discussion will build automatically. There are no wrong answers. Everything you say will be kept confidential.

You can find all the general information in relation to the study design, data collection, and data storage on the information sheet. In addition, we are aiming to find 1-2 women out of this group who have a lot to tell and are willing to be part of an individual interview later. Maybe you can think about that after the discussion.

Is there any question for now? If there are no more questions, I would like to start right away. But please feel free to ask any upcoming question at any point of the interview. I will now start the audio-recording to begin with the first question to be discussed.

*[GBD = gender-based discrimination]*

Interview Questions

1. Could you tell me something about women’s positions in your society? How do they differ from those of men?

- There are many situations in which women might be treated differently than men. In particular, how does this circumstance affect your health and your health behaviors?
- Different treatment because of gender in any situation is generally what we define as GBD. Are you familiar with this expression? If yes, how did you learn about this topic?
- How do you feel talking about GBD and why?

*Objective: To discover existing knowledge and personal perceptions about GBD and to find links and causal pathways between GBD and health.*

1. Have you personally experienced or observed gender-based discrimination? How? In which context?

- Under what circumstances did these experiences happen?
- In your opinion, which of the described situations happen the most and why?
- In your opinion, which of the described situations bother women the most and why?
- If you personally experienced GBD, do you feel that you could ask somebody for help/support? Who?
- In your opinion, what kind of contact points should exist to ask for support?

*Objective: To discover experiences with GBD, how and when these situations occur, to find synonyms for GBD, and to explore existing support systems.*

1. What factors affect your choice to access or seek health care, especially maternal health care?

- Which possible consequences do you think of before seeking health care?
- If there are possibilities to access (maternal) health care facilities, which experiences did you make?
- Do you know women who have changed their behavior after bad experiences when seeking health care?
- What do you think is the general men’s opinion or knowledge about antenatal care?
- Can you tell me about other health issues that you have but the men around you not? Why do they happen to you and/or other women only?

*Objective: To discover the relationship between the lack of accessing maternal health care and GBD, and to discover other indirect causes for health issues based on GBD.*

1. Is there anything else you want to add or something that we have not discussed but seems important to you?

For any question section there can be used the following questions in order to receive additional clarification in the end:

- Can you expand a little on this?
- Can you tell me anything else?
- Can you give me some more examples?
